# Earache and Discharging Ears in Children*Read before Stark county Academy of Medicine, Oct. 19, 1897.

**Published:** 1898-03

**Authors:** Edward P. Morrow

**Affiliations:** Canton, Ohio; Ophthalmologist to the Aultman Hospital


### Selections and Abstracts.

### EARACHE AND DISCHARGING EARS IN CHILDREN.*

By EDWARD P. MORROW, M. D., Canton, Ohio.

Ophthalmologist to the Aultman Hospital.

Beyond the annoyance and suffering endured by the little ones, earache in children is usually looked upon as a trifling affair. When one considers the organ affected and the delicacy of its structures as well as the immense importance of the sense of hearing to the child, then this trouble, instead of being a trifle, assumes importance worthy of study and discussion. Moreover, a large percentage of deaf-mutism can be traced to neglected disease of the middle ear in childhood and infancy. It is the family physician whose attention is first called to these troubles and too often his attention goes no further than to relieve the immediate pains, the subsidence of which dismisses him from the family and the patient from the mind.

In children the common cause of earache is an acute inflammation of the middle ear. In the very young child the trouble is often entirely overlooked until a discharge from the ear announces that the fever and pain have been of local origin in the ear (and not due to spinal meningitis, which it is so often mistaken for and closely simulates). Older children will locate the pain so that the trouble may be early cared for, and if properly treated can often be quickly aborted. It is safe to say that in all cases of recurring earache in children there is a predisposing constitutional cause, the strumous diathesis and inherited syphilis favoring the attacks. Locally as predisposing causes, inspection will reveal enlarged turbinated bodies, deviated septum, thickened pharyngeal walls, adenoids, or enlarged tonsils, or a combination of any or all of these conditions. With these favorable predisposing conditions, every slight attack of cold in the head gives an exciting cause that brings on an attack of middle ear trouble, with the accompanying earache. The epidemic of influenza during the past few winters has added greatly to the number of sufferers from this trouble. Teething is also an exciting cause. The eruption of molar teeth is sometimes preceded by purulent otitis media. It is well in all cases of earache to examine the mouth for de-

* Read before Stark county Academy of Medicine, Oct. 19, 1897.

cayed teeth or roots. In fact the intimate relation of the nervous supply of the teeth and ears should never be overlooked in cases of earache. Another frequent exciting cause is the entrance of cold water externally or through the Eustachian canal. Sea-bathing is a well known cause of many ear troubles, but it is well to know that water at any time is not well borne by the ear. Burnet has said: "It is noteworthy that no mammal but man goes voluntarily under the water without being provided with a means of preventing the water from running into the ears." It is a fact well known that dogs taught to dive become deaf. It is well during the time of the year when children are going into the water to bear these facts in mind. Children should be forbidden to duck the head under water or they should be provided with non-absorbent cotton or wool to keep the water out of the external auditory canal.

Earache occurs also in the course of the acute infectious diseases such as measles, scarlatina, diphtheria, whooping-cough, and in pneumonia, bronchitis and spinal meningitis.

This acute inflammation of the middle ear, causing earache may be either a simple catarrhal inflammation or an acute purulent inflammation, the latter endangering life as well as hearing. The distinguishing points between them are the greater severity of the pain and the height of temperature as early symptoms of the purulent, and after the appearance of the discharge, the purulent character of this. In the catarrhal inflammation the tendency after the attack is to recovery, but neglect of the discharge may and does in many cases result in chronic purulent disease with all of its attending serious results. In the purulent otitis media there are the added dangers of mastoid infection, acute meningitis or brain abscess. As to the treatment of these cases let me say that, as in everything else, early measures are the best. In the simple catarrhal otitis media the disease is often aborted by an early paracentecis of the membrana tympani, preceded and followed by antiseptic precautions in the external canal. Should the disease be well established, the drum membrane bulging and no discharge present, paracentecis should be done at once. This relieves the pain by allowing the pent-up discharges to escape. Following this any mild antiseptic wash may be used sufficiently often to keep the canal thoroughly clean. Should the pain recur it is probable that the opening in the drum membrane has become closed and should be looked after and reopened if necessary. The application to the mastoid of ice or cold water by means of the Leiter coil is grateful and curative. I am sure I need not say that the general condition of the patient should be looked after and treated as indicated. Very often the child's digestion will be found badly out of order and a prescription of

R. Sodii Bicarb....................................................dr. ii
Ext. Rhei Fl.......................................................dr. ii
Tr. Nucis Vomicæ...............................................dr. iss
Aq. Menth. pip, ad.............................................oz. iv
M. Sig. oz. ss to oz. i, t. i. d.

forms a common but very efficient remedy in these cases.

In the purulent inflammation the foregoing treatment is to be carried out so far as the paracentecis is concerned, yet more active measures are necessary in keeping up a local antisepsis. Here the ice applications over the mastoid are very valuable. It is difficult in private practice to carry out the ice applications as they should be, on account of the general prejudice against cold. Next in value are very hot—not warm, but very hot—fomentations made over the mastoid and temporal region. With proper care an acute attack of otitis media will subside in from one day to two or three weeks, according as it is early seen, and the severity of the attack. When once the acute symptoms have subsided, we stand in the presence of what may be a trifling affair, or one of the most serious conditions that can befall a child, namely, a discharging ear. I think I may safely say that the day is forever past when the family physician will advise the parents to allow the discharge to go on. You can all remember when such was the advice. It took years of painstaking investigation and labor to convince the profession that an issue of pus from the ear was a grave matter. Thousands of autopsies have exhibited the extensive damage done to the cranial bones and their precious contents—the brain. I may say thousands of papers have been read to enforce these facts upon the profession, and yet to-day no specialist can help saying that there is still much carelessness on the part of physicians in this matter of discharging ears in children. I say this because I want to give you facts that have been my personal experience. Time and again parents have told me that they have called attention to a discharging ear, to have it turned aside as a matter that either the child would outgrow, or some placebo given to quiet the mother's solicitude. I know well, too, that the laity are at fault in the matter of carelessness in these troubles, but they would not be, once they knew from the right source the serious nature of a chronic otitis media with discharge.

The treatment of a chronic discharge from the ear is never a pleasant one and seldom one upon which to make a reputation. It is only by telling your patient that in all probability the treatment will be long continued and that no one can say what the hearing will be, that it is wise to take a case. Impress upon them that the amount of hearing will be according to the amount of damage already done and that a continuation of the discharge will do more and more harm as the disease continues. Hearing is what the parents are solicitous about, of course. It is hard, very hard, to make them understand that there must be a certain loss of acuteness in hearing in all cases where the ear has been affected for any length of time. The discharge does not much trouble the parents, the loss of hearing does. The discharge they become accustomed to. The loss of hearing alarms them, and it is only the intelligent who seem to associate the two. Of still greater gravity than the loss of hearing is the danger to life in these chronic discharging ears. You, who are examiners for insurance companies, know that they will not assume a risk in a case of chronic discharge from the ear, and look askance upon one who has ever had a discharging ear. The involvement of the mastoid cells in this trouble may well be termed one of the grave diseases.

It is not the acute mastoiditis that is the most serious, but the chronic that creeps insidiously along for years, that results so often fatally. Sometimes the cause of death is made known only after opening the cranium and finding the abscess. I call to mind the case of a young man where I used all of my influence upon the patient and family in conjunction with the family physician to urge an operation in a case of chronic mastoiditis. He had had a discharging ear from childhood. When I saw him he was about twenty years of age and manifested all of the symptoms of chronic mastoiditis. Within six months he died in convulsions, the result of probable abscess of the brain.

The family physician and myself had already been dismissed from the case. The physician who attended him gave me a history of his symptoms, but had failed to make a proper diagnosis of the trouble, because the parents assiduously kept from him the fact that he had a chronic ear disease, fearing, no doubt, that an operation would again be proposed.

It may be well at this point to speak of the gravity of the mastoid operation. The operation in itself is not a grave one. Very few cases are recorded where death can be traced to the operation, even although the cranial cavity has been accidentally entered. An unfavorable termination following an operation usually depends upon the extensive involvement found at the time and can in no way be traced to the procedure adopted for its relief. I find in most of the statistics upon the subject that death is occasioned by intracranial involvement before the operation has been undertaken. In most cases the operation is so gratifying in its results that a cure may be promised. In acute cases in children a deep incision down to the mastoid, known as Wilde's incision, is admirable and may be made before a more extensive operation is undertaken. In any instance an early operation gives the best results.

I have endeavored to trace from a common, every-day earache in the child the possibility of such a grave outcome as mastoiditis. I have spoken of the operation, but better than any operation and indicative of greater skill is the prevention of the causes leading to this trouble. I do not presume to say that all or nearly all of these discharging ears can be seen by a physician early enough to prevent all serious trouble, but I do say that with our present knowledge the physician can overcome to a great extent the neglect of children who suffer earache and have chronic otorrhea.—*American Therapist.*

